# N-acetyl-L-cysteine reduces Leishmania amazonensis-induced inflammation in BALB/c mice

**DOI:** 10.1186/s12917-020-2234-9

**Published:** 2020-01-13

**Authors:** Rosalia Crupi, Enrico Gugliandolo, Rosalba Siracusa, Daniela Impellizzeri, Marika Cordaro, Rosanna Di Paola, Domenico Britti, Salvatore Cuzzocrea

**Affiliations:** 1 Department of Chemical, Biological, Pharmaceutical and Environmental Sciences, University of Messina, Viale Ferdinando Stagno D’Alcontres n°31, 98166 Messina, Italy; 2 Department of Veterinary Science, University of Messina, Messina, Italy; 3 a C.I.S. - Interdepartmental Services Centre of Veterinary for Human and Animal Health, Magna Graecia University of Catanzaro, Catanzaro, Italy; 4 Department of Pharmacological and Physiological Science, Saint Louis University School of Medicine, Saint Louis, USA

**Keywords:** Leishmaniasis, Inflammation, Mice, Pain

## Abstract

**Background:**

Leishmaniasis is a emergent disease characterized by different clinical manifestations in both humans and dogs. Predominant clinical features of cutaneous leishmaniasis are ulcerative painless skin lesions. Several data reported that pain is associated with human and dog leishmaniasis, out with areas of painless ulcerative lesions per se. Actually, current medications used for leishmaniasis management are characterized by several side effects and, in addition, some cases of the disease are refractory to the treatment. On this background it is mandatory the identification of new and safe candidates for designing less toxic and low-cost remedies. Therefore, the search for new leishmanicidal compounds is indispensable.

**Methods:**

In the present paper we investigated the effect of orally N-acetyl-L-cysteine (NAC) supplementation at dose of 200 mg/Kg for 10 weeks, in subcutaneous *Leishmania* (L). *amazonensis* infected BALB/c mice. And evaluating the effect of NAC on inflammatory response such as TNF-α, IL-6, IL-1β levels, and on thermal and mechanical hyperalgesia.

**Results:**

In the present paper we showed how NAC supplementation affected parameters of oxidative stress (GSH, MDA, SOD), inflammation such as cytokines levels (IL-1β, IL-6, TNFα) and mast cell activation and consequently on induced pain, during leishmaniosis in BALB\c mice.

**Conclusions:**

The findings of our study provided the scientific data demonstrating that *L. amazonensis* infection induces inflammation and pain in BALB/c mice that are reversed by administration of NAC.

## Background

Leishmaniasis, a severe public health problem worldwide, is proved by other than 20 various protozoan species of the genus Leishmania [1]. This disease represents one of the most frequently occurring painful and inflammatory conditions in both humans and animals [2, 3]. These parasites are classified as digenetic organisms because they live one phase of their lifecycle in an insect host from the genus Lutomzyia in the New World or Phlebotomus in the Old World and the other stage inside a mammalian host. A wide umbrella of clinical manifestations characterized leishmania infections and are recognized by distinctive symptoms principally related to infections and diverse Leishmania species [4]. The different types of the disease have been categorized in tegumentary, also named cutaneous leishmaniasis, and visceral leishmaniasis. About 200,000–400,000 new visceral leishmaniasis cases and 700,000– 1,200,000 new cutaneous leishmaniasis cases happen each year worldwide [5]. The transmission of parasites occur by phlebotomine sand flies which become infected during blood meal on mammalian hosts. Though dogs represent the most know domestic reservoirs of specific Leishmania parasites such as *L. infantum* the role of Didelphis spp. as reservoirs have been proposed and its synanthropic facility could simplify the link between wild and peridomestic environments [6, 7]. Literatura data showed that various rodent species have been classified as probable pools of *L. amazonensis*, *L. braziliensis* and *L. infantum*, screening competence to maintain these parasites [8]. Because of this, leishmaniasis is classified as a re-emergent and new infection with geographical extension due to human migration, urbanization, human-driven environmental alterations and co-infection with other illnesses [9]. The important expansion of this disease has pointed the development of new foci of transmission and reactivation in controlled sets [10, 11]. In the spectrum of Leishamnia parasites, the L. amazonensis belongs to the *L. mexicana* complex and produces localized cutaneous and diffuse cutaneous leishmaniasis [5]. This particular parasite frequently capture the host’s innate immune response to start an infection, in particular in macrophages. The infections caused by *L. amazonensis* are featurized by the suppression of the natural early response, as reported by inhibition of macrophage production of pro-inflammatory molecules. It was demonstrated that in the in the initial stage of infection by *L. amazonensis*, many inflammatory cytokines are downregulated when matched with *L. major* infected mice [12]. Others reports demonstrated that the suppression of pro-inflammatory cytokines such as IL-12, IL-17 and IL-6 in macrophages infected with *L. amazonensis* and preserved with lipopolysaccharide (LPS) as compared with infection by *L. major* [13]. Clinical manifestation of the disease comprise a widespread range of expressions, among these skin ulcers at the set of the infection or distribution in visceral organs followed by leucopenia, anemia, weakness and fever [14]. Actually there is no efficient vaccine against leishmaniasis and also chemotherapy comprises restricted pletora of drugs. The pharmacological treatment employed to counteract the effects of infections showed several difficulties such as high toxicity and cost, chronic administration, inconsistency of efficiency and parasite resistance [15]. For these reasons the elaboration of new current and also reasonable drugs is essential. In the pathophysiology of leishmania a prominent role is coated by both inflammation and oxidative stress.. The relationship between inflammation and the production of reactive species is well known and characterized as a fundamental component of oxidative stress, the oxidative stress often represents an additional component to the tissue damage induced by leishmaniasis. Reactive oxygen species (ROS) are generated in the mitochondria reported as respiratory chain products [16] and are implicated in several biological processes, such as hormonal biosynthesis [17], cellular signalling [18] and destruction of intracellular pathogens [19]. Moreover, ROS are also classified as relevant effector agents against intracellular pathogens, induced by Toll-like receptors or IFN-γ [20, 21]. Data obtained from investigations carried out in murine model of leishmaniasis proved that nitric oxide (NO) induction is one of the key effector mechanisms of macrophages for abolition of Leishmania parasites [22]. Researches data suggests that the susceptibility of a selected strain of mice such as BALB/c mice to *L. amazonensis* as well recognized to *L. major* infection appears to be dependent of Th2 cytokines like IL-4 and IL-10 [23]. Currently, the most frequently used drug for dogs are systemic antimonials [24]. Nevertheless, because of the high toxicity related with this group and the recent emergence of drug resistant strains, alternative therapeutic options to be considered. Oxidative stress is considered to be an important etiologic factor in leishmaniasis and the antioxidant NAC has been used with success as an adjunct in human and experimental leishmaniasis diseases [25]; formulations of NAC have utilized, both in the human [24] and veterinary fields [26]. NAC is classified as an antioxidant, both directly as a GSH substitute and indirectly as a precursor for GSH [27]. Therefore, research is now focused on the identification of more effective and safe tools, as part of an ideal multimodal management of leishmaniosis and its complications in veterinary patients. On this basis, we investigated the possible effects of NAC supplementation on the course of *L. amazonensis* infection in BALB/c mice to better clarify both pathogenesis and development of leishmaniasis. In particular we focused our research on the inflammatory process and consequently algesic sensitization associated with leishmaniosis disease. We used a multimodal approach evaluating the inflammatory response and the involvement of important immune cells such as mast cell in pain sensitization, and also the antioxidant activity of NAC. Our study confirms and suggest an important role for NAC as a useful strategy or coadjuvant in the treatment of this multi symptomatic and complex disease.

## Results

### NAC decreases paw edema, histological damage and neurotrophilic infiltration in *L. amazonensis*-infected animals

**Fig. 1:**
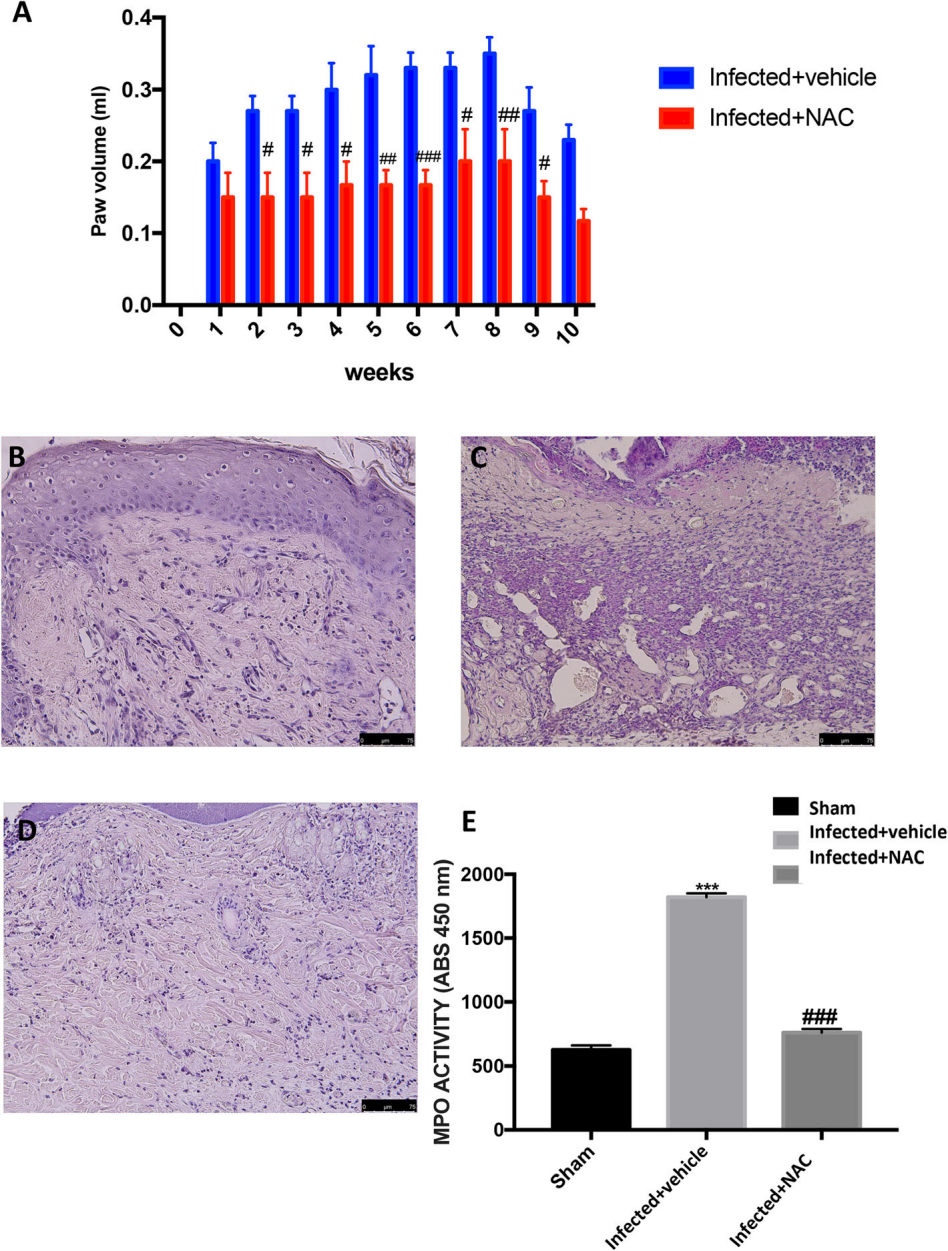
Effect of oral NAC on *L. amazonensis* infected mice on paw edema histological damage and neutrophil infiltration in paw skin. Paw edema (**A**) was assessed at the time points indicated after subcutaneous injection of *L. amazonensis* into the mice hind paw. NAC administration (200 mg/kg) produced significant improvements in this scores. Histological analysis was achieved by hematoxylin/eosin staining in sham; (**B**), *L. amazonensis* subcutaneously infected mice into the hind paw (**C**), and *L. amazonensis* subcutaneously infected mice + NAC (200 mg/kg) (**D**); moreover myeloperoxidase (MPO) activity in paw skin was evaluated (**E**). Figures are representative of at least three independent experiments for all animals from each group. Values are means ± SEM of five animals for each group. ^###^*P* < 0.001 vs. *L. amazonensis* infected mice; ^***^P < 0.001 vs. sham

The inflammatory response in the paw skin revealed a significant difference in infected animals when matched to the treated-infected animals. Oral administration of NAC (200 mg/kg) significantly reduced the development of paw edema beginning, namely from the second week (Fig. 1a). Skin from sham mice appeared normal (Fig. 1b). In contrast, a evident increase of inflammatory cells was evident after *L. amazonensis* infection into the right hind paw (Fig. 1c). Orally NAC administration (200 mg/kg) significantly reduced this histological alteration, as well as inflammatory cell infiltration (Fig. 1d).

Histological injury progression was also linked with neutrophil infiltration revealed by an increase in MPO activity (Fig. 1e).

The infection with *L. amazonensis* stimulated significant increase in MPO compared with control, whereas treatment with NAC (200 mg/kg) notably reduced MPO activity when compared to vehicle (Fig. 1e).

### Effect of NAC on chronic mechanical and thermal hyperalgesia in *L. amazonensis* infected animals

**Fig. 2:**
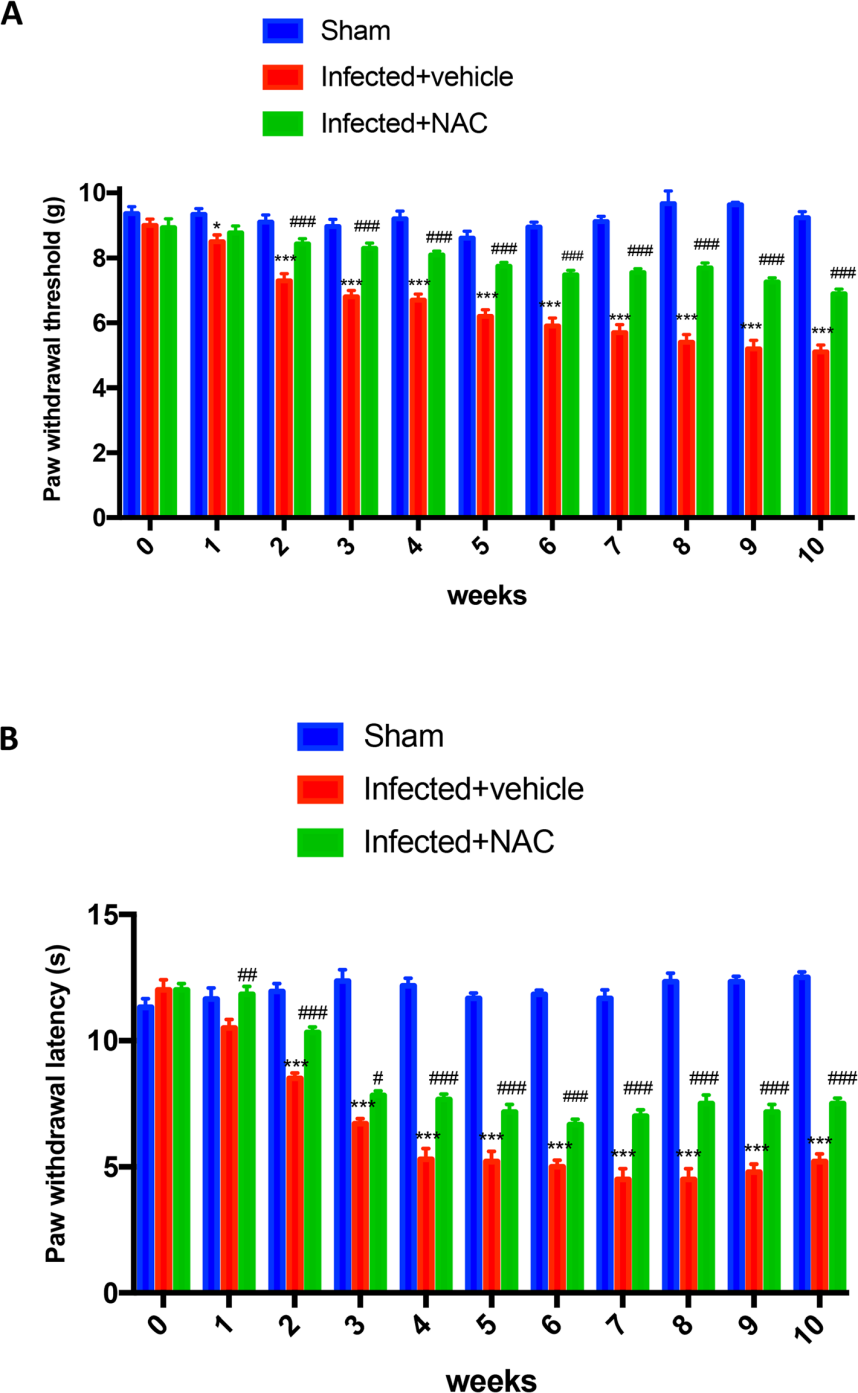
Effect of oral NAC administration on *L. amazonensis* infected mice on mechanical and thermal hyperalgesia. Mechanical (**A**) and thermal hyperalgesia (**B**) were assessed at the time points indicated after subcutaneous infection of *L. amazonensis* into the mice hind paw. NAC administration (20 mg/kg) produced significant improvements in both scores. Values are means ± SEM ^#^*P* < 0.05, ^##^*P* < 0.01, and ^###^*P* < 0.001 vs. *L. amazonensis* infected mice

Subsequently we evaluated the effect of NAC on mechanical and thermal hyperalgesia. Oral administration of NAC (200 mg/kg) significantly reduced the development of mechanical and thermal hyperalgesia starting from the second week (Fig. 2b, c).

### Effect of NAC on mast cell infiltration and nociceptive marker in *L. amazonensis* infected mice

**Fig. 3:**
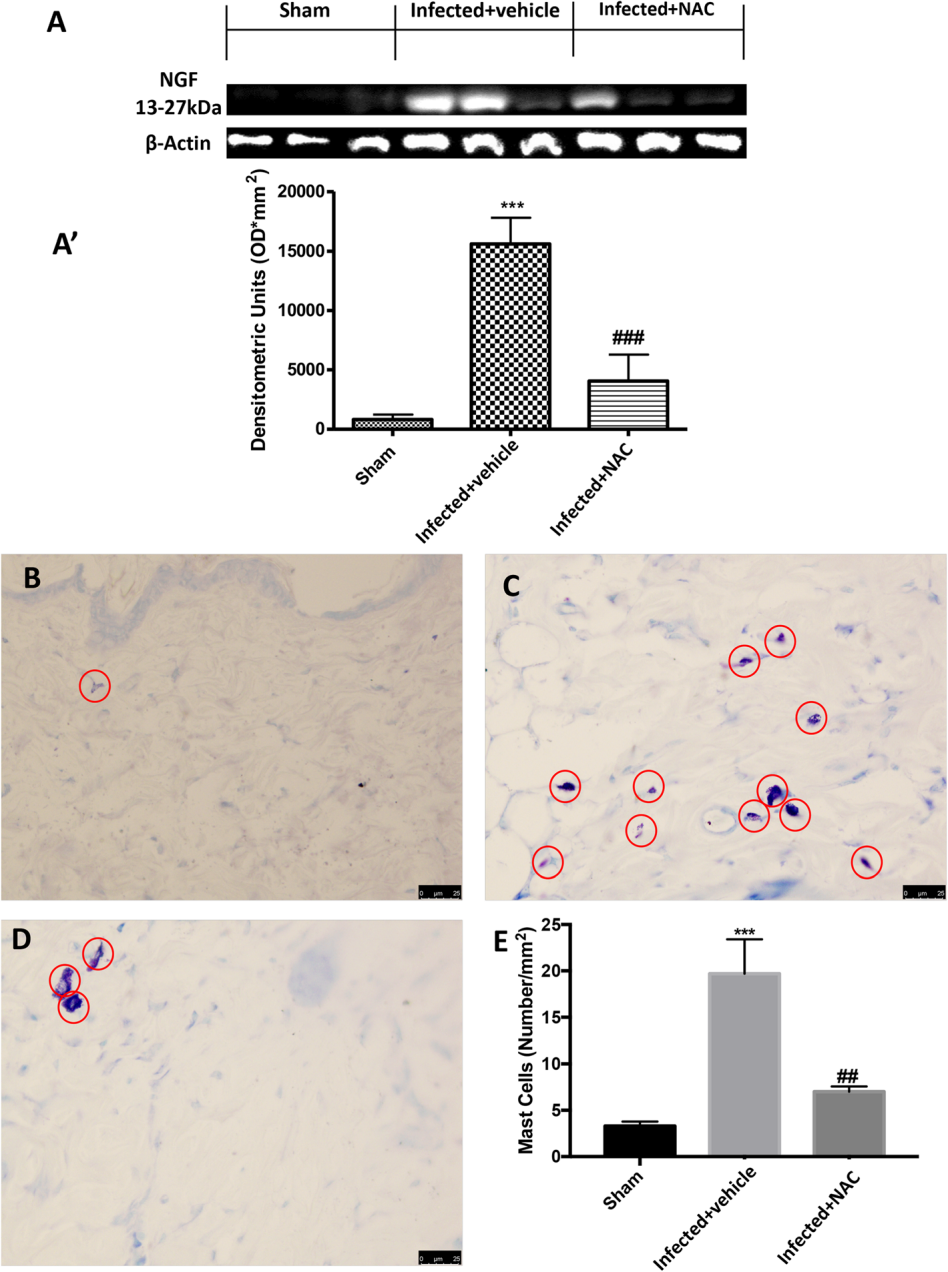
Effect of oral NAC administration on *L. amazonensis* infected mice on NGF and mast cell infiltration in paw skin. Representative western blot showing the effect of NAC (200 mg/kg) on NGF (**A, A’**). Levels of NGF presented in the densitometric analyses of protein bands were normalized for β-actin. The number of mast cell was increased in *L. amazonensis* infected mice (**C**) compared to the sham animals (**B**). Administration of NAC (200 mg/kg) (**D**) reduced mast cells infiltration in paw skin; the mast cells score was done by independent observer (**E**). Data are representative of at least three independent experiments and are expressed as mean ± SEM from *N* = 5 mice/group. A representative blot is shown and densitometric analysis is reported. All figures are representative of at least three independent experiments for all animals from each group. Values are means ± SEM ^#^*P* < 0.05, ^##^*P* < 0.01, and ^###^*P* < 0.001 vs. *L. amazonensis* infected mice

Level expression of the nerve sensitizer NGF (Fig. 3a,a’) was increased in the *L. amazonensis* infected animals matched to sham animals. Administration of NAC (200 mg/kg) significantly counteracted such increase (Fig. 3a,a’). *L. amazonensis* infected mice paw skin, stained with toluidine blue, showed a strong infiltration of mast cells (Fig. 3c,e), as compared to sham mice (Fig. 3b,e). Orally NAC administration (200 mg/kg) significantly reduced mast cell infiltration (Fig. 3d,e).

### NAC reduces cytokine release in *L. amazonensis* infected animals

**Fig. 4:**
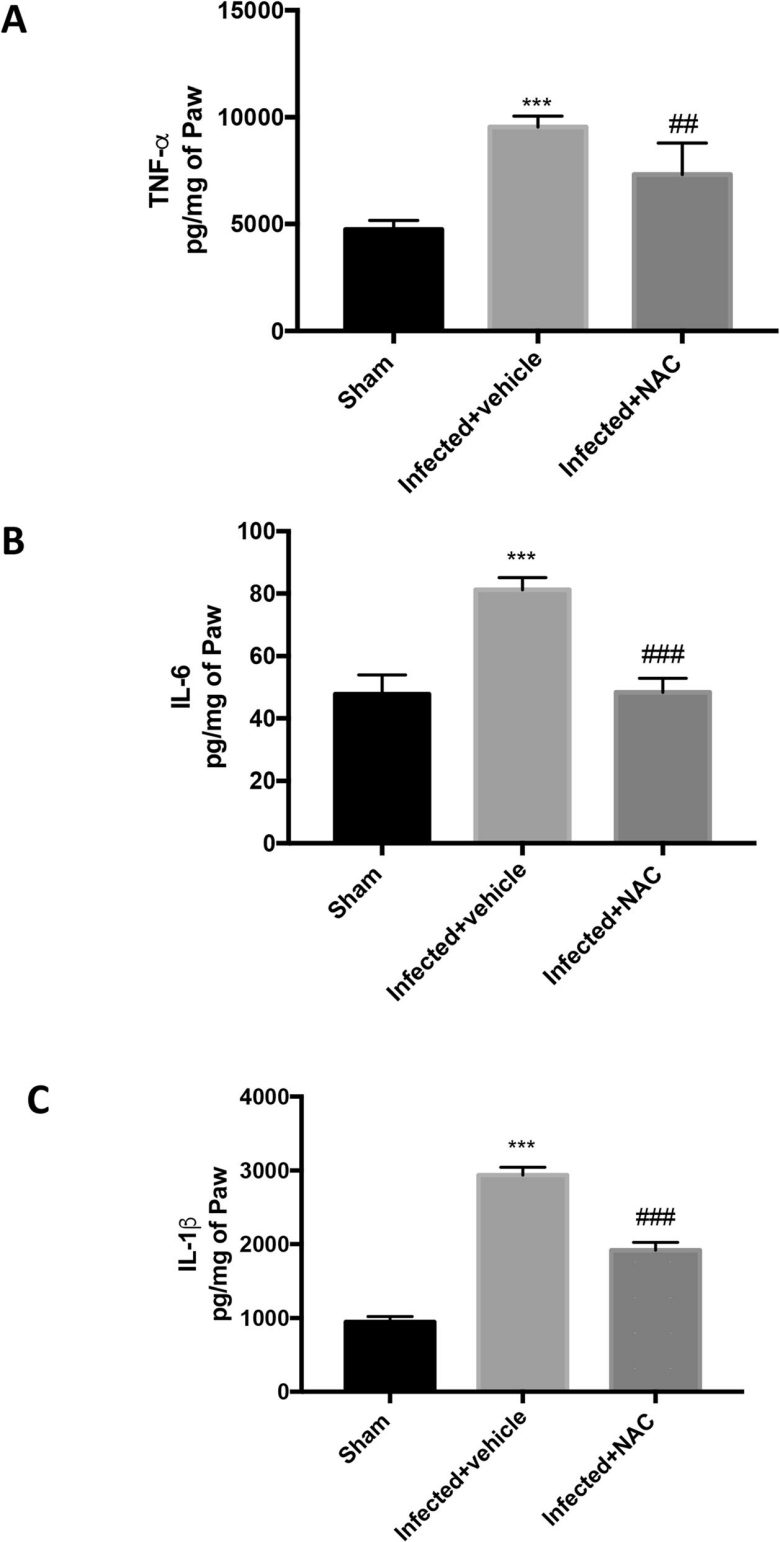
Effect of oral NAC administration on *L. amazonensis* infected mice on cytokine expression. A significant increase of TNF-α (**A**), IL-6 (**B**), and IL-1β (**C**) levels was detected in paw skin after subcutaneosus infection of L. amazonensis into the mice paw skin. Orally NAC administration (20 mg/kg) significantly reduced levels of all analized cytokines. Values are means ± SEM of five animals for each group. ^###^*P* < 0.001 vs. L. amazonensis infected mice; ^***^P < 0.001 vs. sham

The decrease of mice paw edema, mechanical and thermal hyperalgesia after NAC administration (200 mg/kg) was related with a significant reduction in paw skin content of pro-inflammatory and pro-nociceptive cytokines such as TNF-α (Fig. 4a), IL-6 (Fig. 4b) and IL-1β (Fig. 4c), as matched to the *L. amazonensis*-infected mice.

### Effect of NAC on oxidative stress markers alteration in *L. amazonensis* infected animals

**Fig. 5:**
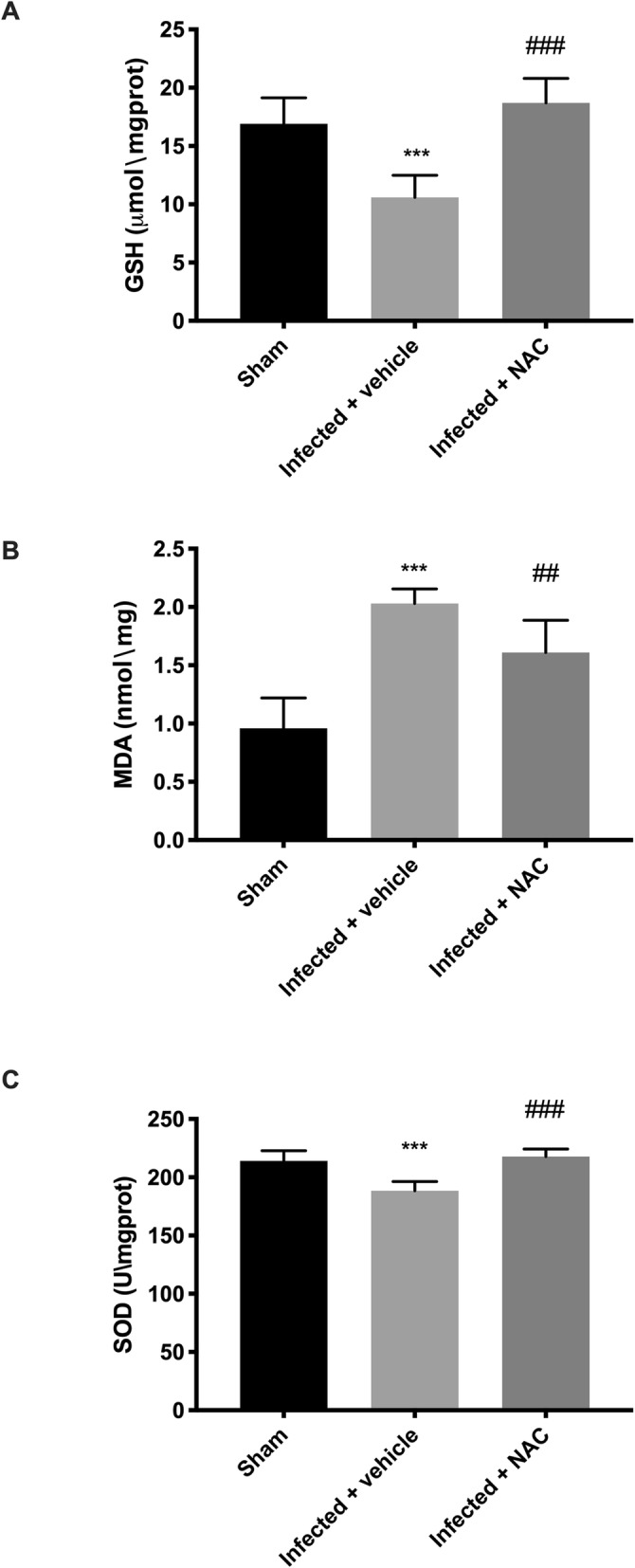
Effect of NAC on oxidative stress markers alteration in *L. amazonensis* infected animals. As show in Fig. 5**A** in infected + vehicle mice we observed a significant depletion of GSH levels, that have been restored by NAC treatment. The result in Fig. 5**B**, showed that the level of MDA was decreased significantly in tissue collected from mice treated with NAC compared to *L. amazonensis +* vehicle infected animals. Furthermore, we observed that the activities of SOD Fig.5**C**, were significantly increased in tissue taken from NAC group treated compared to infected + vehicle mice group. Values are means ± SEM of five animals for each group. ^##^*P* < 0.01,^###^*P* < 0.001 vs. L. amazonensis infected mice; ^***^P < 0.001 vs. sham

To evaluate the antioxidant effect of NAC, we investigated some markers of oxidative stress, such as glutathione (GSH), malondialdehyde (MDA) level and superoxide dismutase (SOD) activity, as show in Fig. 5a in infected + vehicle mice we observed a significant depletion of GSH levels, that have been restored by NAC treatment. As illustrated in Fig. 5b, the result showed that the level of MDA was decreased significantly in tissue collected from mice treated with NAC compared to *L. amazonensis +* vehicle infected animals. Furthermore, we observed that the activities of SOD Fig.5c, were significantly increased in tissue taken from NAC group treated compared to infected + vehicle mice group.

## Discussion

In the present research we have verified whether and how orally NAC act an anti-inflammatory antioxidant and conseguently an analgesic effect in subcutaneous *L. amazonensis*-infected BALB/c mice, in an attempt of confirming the impact of this treatment in both pathogenesis and development of leishmaniasis disease. *L. amazonensis* is the contributory cause of cutaneous and cutaneous diffuse leishmaniasis; in both forms of infection chronic lesions are disseminated through the skin and for this reason are classified are disabling disease with difficult management. Moreover, several patients refer pain independently of the region of the body infected [28]. The inflammatory process related with infection is also related with pain condition. This symptom is due to the development of immune response including inflammatory mediators formation, leading to nociception sensitization of primary and second-order neurons implicated in nociceptive impulse transmission. Nevertheless, these processes cause central sensitization and therefore the sensation of pain perceived as hyperalgesia [29, 30]. The inflammatory process includes, among others, a severe involvement of cytokines participating in the determination of pain. Literature data with *Leishmania* parasites encourage the development of chronic inflammatory lesions associated with cytokines release that provoke tissue damage and pain [31, 32]. It was demonstrated that chronic *L. amazonensis* infection in BALB/c mice displays induction of inflammatory response in particular in the site of infection; this inflammatory condition is also associated with hyperalgesia, parasite migration and injury in secondary organs like liver and spleen [33, 34]. Moreover, a nociceptive behavior was seen in both rats and mice infected with *L. major* and *L. amazonensis*, despite absence of pain was reported in patients with cutaneous lesions [33, 35]. In the field of inflammatory process, cytokines such as IL-1b, TNF-a and IL-6 have a role as hyperalgesic endogenous molecules and analgesic targets [36]. Leishmania parasite resides within macrophages and may cause inhibition of mitochondrial respiratory activity, also inactivates peroxidases, increases susceptibility to oxidant damage, inhibits glycolysis, S-nitrosylation, ADP-ribosylation, nitrosine tyrosine of proteins, disrupts the Fe-S clusters, zinc fingers or heme groups and causes peroxidation of membrane lipids [37]. Still many of the pathogenetic mechanisms of leishmaniasis are not clear, as well as the interactions with the immune system and the host resistance mechanisms. An important role is attributed to macrophages, in fact the recruitment and the activation of macrophages can influence the outcome of Leishmania infection, the activated macrophages are also responsible for the production of high reactive species qualities of nitrogen and oxygen during oxidative burst, even if some species are resistant to this oxidative stress [38].. This type of parasite resistance probably developed as a defense mechanism against the host’s response, moreover, this type of resistance of the parasite stimulates a continuous and intense inflammatory response by the host. Therefore the inflammatory response and the continuous production of oxidizing agents during this phase contributes to tissue injury and therefore to disease progression [39]. Is widely recognized that oxidative stress and oxidative damage to biomolecule are involved in the progression of many diseases, especially in association with pro inflammatory conditions. This has been seen not only in infectious diseases but in a whole series of pathologies of various nature such as cardiovascular conditions, several types of cancer, neurodegenerative diseases and diabetes, where the major regulator of the pathological process is the activation at the molecular level of a pro-inflammatory condition [39, 40]. The current therapies for leishmaniasis are all characterized by important side effects as well as by toxicity if used for long periods [41, 42]. In light of the relationship between activation of pro-inflammatory pathways and production of reactive species, oxidative stress is an additional factor that contributes to tissue damage caused by leishmaniasis, and therefore to be taken into consideration as a pharmacological target. Our data showed a structural abnormalities and neurotrophilic infiltration in skin of *L. amazonensis* infected mice and that in terms of paw edema, thermal and mechanical hyperalgesia, neutrophilic infiltration and skin damage. NAC also counteracted the L. amazonensis -induced output of pro-inflammatory and pro-nociceptive mediators in paw skin. It is well known that mast cells play an important part both in physiological and pathological situations, since they are able to release different proinflammatory mediators and thus increase inflammation of leukocytes in inflammatory states. It is known that the combination of oxidative stress and inflammatory activation is commonly associated to several degenerative processes triggered by parasitosis. It was also seen as TNF-α promotes the induction of IL-6 and IL-1β; high levels of these pro-inflammatory cytokines can cause systemic inflammation [43]. Glutathione (GSH) and the other proteins containing thiol groups are the main endogenous components responsible for protection from oxidative stress, in fact they have the ability to reduce the excess of electrophilic species that would otherwise cause damage to biomolecules. GSH is primarily responsible for the detoxifying action of liver, by both xenobiotics and endogenous metabolites, which are thus eliminated by being conjugated with GSH. In response to oxidative stress, GSH and other proteins containing thiol groups are up regulated, due to their action capable of to scavenge superoxide and hydroxyl radicals [44] [45]. The protective effects of NAC have been suggested to be due to scavenging reactive oxygen intermediates thought a stimulation of glutathione synthesis [46] [45], which modulates the immune response [47].

## Conclusions

In conclusion, the present findings demonstrate that L. amazonensis infection induces inflammation, pain and oxidative stress in BALB/c mice. In particular, in this study we have shown that BALB/c mice subjected to infection with *L. amazonensis* exhibit an increase in paw edema associated with histological damage and neutrophilic infiltration.

Chronic treatment with NAC decreased inflammation, pain and the levels of the principal pro-inflammatory cytokines such as IL-1β, IL-6 and TNF-α. Based on our results, a clinical use of NAC as an adjuvant to current therapies against leishmaniasis would seem to be of great help in the management of this complex pathology, which could help to prevent the development of disorders resulting from the association between inflammation, pain and oxidative stress. These preclinical findings contribute to address significant questions related to biological events observed in human leishmaniasis and may represent great advances in the complex understanding about the evolution of lesions in cutaneous leishmaniasis. Although, the limitation of preclinical models on the transferability of efficacy in veterinary medicine, the present findings shed new light on some of the inflammatory and nociceptive pathways and molecules targeted by NAC. To date there is an increasing interest in the search for new drugs for the treatment of leishmaniasis, which can also have a different mechanisms of action from those that are the currently available drugs. Collectively the data presented in our study suggest an important role of NAC in the management of pain and especially of the inflammatory process in leishmaniasis, therefore NAC could be a great resource for the management of these pathologies in veterinary medicine.

## Methods

### Animals

Male BALB/c mice (6–8 weeks old; 25–30 g) were purchased from Envigo (Milan, Italy) and were housed in a controlled location and delivered with typical rodent water and food. Mice were acclimatized to their environment for 1 week. Animal care was in accord with Italian (DM 116192) and European Economic Community regulations (OJ of EC L 358/1 12/18/1986) for the protection of experimental animals. Animals study was conducted according to ARRIVE guidelines.

### Parasites and mice infections

*L. amazonensis* (WHOM/R/75/Josefa) was cultivated in vitro in Schneider Insect Medium (Sigma-Aldrich) added with 10% FBS and 100 U/ml penicillin and 100 mg/ml streptomycin. Stationary promastigote forms were transferred to fresh medium at a density of 10^7^ parasites/ml, at 26 °C. BALB/c mice were subcutaneously infected in the footpad with 2 × 10^5^ stationary phase promastigotes of *L. amazonensis,* as previously described [48].

### Experimental groups and treatment

Animals were randomly assigned into the following groups:

Sham (non-infected animals): mice (*N* = 10) were exposed to same surgical procedures excluding for the infection with parasite.

Infected + vehicle: mice (*N* = 10) were subcutaneously infected in the footpad with 2 × 10^5^ stationary phase promastigotes of *L. amazonensis.*

Infected + NAC: mice (*N* = 10) were administered 2 days before the infection with NAC 200 mg/kg once a day during all the infection period (10 weeks) by oral gavage as previously seen [48].

At the end of experiment, animals were terminally anesthetized with overdose of isoflurane (5.0% isoflurane (Baxter International) in air, until saturation) for further analysis.

The minimum number of animals for each technique was calculated with G*Power software statistical test. This test provides an efficient method to determine the sample size necessary to carry out the experiment before the experiment itself is actually conducted.

### Paw edema

Volume of paw edema was calculated by quantitating the variation in the paw volume using a plethysmometry (model 7140; Ugo Basile).

### Histological analysis and myeloperoxidase (MPO) activity

Sections of 7 μm-thick were stained with haematoxylin and eosin and then observed by light microscopy coupled to an Imaging system (Leica DM2000, Milan, Italy) The MPO activity, an indicator of neutrophil infiltration, was made and calculated as previously described [49].

### Thermal hyperalgesia

The responses to thermal hyperalgesia was calculated by the Hargreaves’ Method using a Basile Plantar Test [50] (Ugo Basile; Comeria, Italy) with a cut-off latency of 20 s employed to prevent tissue injury. The withdrawal latency time of irritated paws was calculated with an electronic clock circuit and thermocouple. Foot withdrawal latencies were acquired before infection (baseline) and after every week for all experimental time to calculate the analgesic effect of NAC treatment. A significant (*P* < 0.05) decrease in paw-withdrawal latency over time is described as thermal hyperalgesia.

### Mechanical Hyperalgesia

Mechanical allodynia was measured by Electronic von Frey test (BIO-EVF4, Bioseb, France). The withdrawal threshold was expressed as the force, in grams, at which the mouse withdrew its paw. Withdrawal was determined 3 times, and the reported value is the mean of the 3 different measures.

### Western blot analysis

Western blot analysis on cytosolic fraction of the paw skin was made as previously described [51]. The filters were probed with anti-nerve growth factor (NGF) (1:1000) (Santa Cruz Biotechnology Cat# sc-549, RRID:AB_632012) and β-actin (1:500) (Santa Cruz Biotechnology Cat# sc-69,879, RRID:AB_1119529) for the standardization. Signals were detected with enhanced chemiluminescence (ECL) detection system reagent and the relative expression of the protein bands was quantified by densitometry with BIORAD ChemiDocTM XRS + software. A representative picture of blot signals was imported to analysis software (Image Quant TL, v2003).

### Staining of mast cells (MCs)

The identification of MCs was evaluated in the palm of paw sections as previously described by Petrosino et colleagues [52]. Briefly, sections were detected with toluidine blue and the mast cells were marked purple. Metachromatically stained MCs were calculated by counting five high-power fields (100×) per section using Leica DM2000 (Milan, Italy) microscope.

### Determination of cytokine levels in paw skin

TNF-α, IL-1β and IL-6 released in the paw skin were measured by ELISA (R&D systems, Minneapolis, MN) as described previously by Salvemini et al. [53], and the results expressed as pg per paw normalized to the volume recovered from each paw.

### Assay of oxidative stress

Assay of oxidative stress was performed as previously described [54–56]. MDA level, SOD activity, GSH level were spectrophotometrically analyzed according to the manufacturer instructions.

### Statistical analysis

All values are expressed as mean ± standard error of the mean (S.E.M.) of N observations. Each experiment was performed in triplicate on three different occasions. All analysis was executed in a blinded manner. The significance level was determined by one-way analysis of variance (ANOVA). GraphPad Software Prism 7 (La Jolla, CA) was used for all statistical analyses.

## Data Availability

The datasets generated and analyses during the current study are available from the corresponding author on reasonable request.

## Abbreviations

ECL: Enhanced chemiluminescence
GSH: Glutathione
L. amazonensis: Leishmania amazonensis
LPS: Lipopolysaccharide
MCs: Mast Cells
NAC: N-acetyl -L-cysteine
NGF: Nerve growth factor
S.E.M.: Standard error of the mean

## References

[1] Alvar J, Velez ID, Bern C, Herrero M, Desjeux P, Cano J, Jannin J, den Boer M, Team WHOLC (2012). Leishmaniasis worldwide and global estimates of its incidence. PLoS One.

[2] Burza S, Croft SL, Boelaert M (2018). Leishmaniasis. Lancet.

[3] Ceron JJ, Pardo-Marin L, Caldin M, Furlanello T, Solano-Gallego L, Tecles F, Bernal L, Baneth G, Martinez-Subiela S (2018). Use of acute phase proteins for the clinical assessment and management of canine leishmaniosis: general recommendations. BMC Vet Res.

[4] Murray HW, Berman JD, Davies CR, Saravia NG (2005). Advances in leishmaniasis. Lancet.

[5] Silveira FT, Lainson R, Corbett CE (2004). Clinical and immunopathological spectrum of American cutaneous leishmaniasis with special reference to the disease in Amazonian Brazil: a review. Mem Inst Oswaldo Cruz.

[6] Santiago ME, Vasconcelos RO, Fattori KR, Munari DP, Michelin Ade F, Lima VM (2007). An investigation of Leishmania spp. in Didelphis spp. from urban and peri-urban areas in Bauru (Sao Paulo, Brazil). Vet Parasitol.

[7] Carreira JC, da Silva AV, de Pita PD, Brazil RP (2012). Natural infection of Didelphis aurita (Mammalia: Marsupialia) with Leishmania infantum in Brazil. Parasit Vectors.

[8] Roque AL, Jansen AM (2014). Wild and synanthropic reservoirs of Leishmania species in the Americas. Int J Parasitol Parasites Wildl.

[9] Desjeux P (2004). Leishmaniasis: current situation and new perspectives. Comp Immunol Microbiol Infect Dis.

[10] Barata RA, Peixoto JC, Tanure A, Gomes ME, Apolinario EC, Bodevan EC, de Araujo HS, Dias ES, Pinheiro Ada C (2013). Epidemiology of visceral leishmaniasis in a reemerging focus of intense transmission in Minas Gerais state Brazil. Biomed Res Int.

[11] Arce A, Estirado A, Ordobas M, Sevilla S, Garcia N, Moratilla L, de la Fuente S, Martinez AM, Perez AM, Aranguez E (2013). Re-emergence of leishmaniasis in Spain: community outbreak in Madrid, Spain, 2009 to 2012. Euro Surveill.

[12] Ji J, Sun J, Soong L (2003). Impaired expression of inflammatory cytokines and chemokines at early stages of infection with Leishmania amazonensis. Infect Immun.

[13] Lapara NJ, Kelly BL (2010). Suppression of LPS-induced inflammatory responses in macrophages infected with Leishmania. J Inflamm (Lond).

[14] Martinez E, Torres-Guerrero E, Cortes E, Tejada D, Arenas R (2017). Cryptococcus laurentii infection in a patient with cutaneous leishmaniasis. Int J Dermatol.

[15] Colhone MC, Silva-Jardim I, Stabeli RG, Ciancaglini P (2015). Nanobiotechnologic approach to a promising vaccine prototype for immunisation against leishmaniasis: a fast and effective method to incorporate GPI-anchored proteins of Leishmania amazonensis into liposomes. J Microencapsul.

[16] Rada B, Leto TL (2008). Oxidative innate immune defenses by Nox/Duox family NADPH oxidases. Contrib Microbiol.

[17] Chan YC, Leung PS (2011). The renin-angiotensin system and reactive oxygen species: implications in pancreatitis. Antioxid Redox Signal.

[18] Landry WD, Cotter TG (2014). ROS signalling, NADPH oxidases and cancer. Biochem Soc Trans.

[19] Nusse O (2011). Biochemistry of the phagosome: the challenge to study a transient organelle. ScientificWorldJournal.

[20] Kavoosi G, Ardestani SK, Kariminia A (2009). The involvement of TLR2 in cytokine and reactive oxygen species (ROS) production by PBMCs in response to Leishmania major phosphoglycans (PGs). Parasitol.

[21] Pawate S, Shen Q, Fan F, Bhat NR (2004). Redox regulation of glial inflammatory response to lipopolysaccharide and interferongamma. J Neurosci Res.

[22] Liew FY, Xu D, Chan WL (1999). Immune effector mechanism in parasitic infections. Immunol Lett.

[23] Guimaraes ET, Santos LA, Ribeiro dos Santos R, Teixeira MM, dos Santos WL, Soares MB (2006). Role of interleukin-4 and prostaglandin E2 in Leishmania amazonensis infection of BALB/c mice. Microbes Infect.

[24] Al-Natour SH (2009). Update in the treatment of cutaneous leishmaniasis. J Family Community Med.

[25] Oliveira E, Saliba JW, Oliveira D, Dias ES, Paz GF (2016). A prototype of the direct agglutination test kit (DAT-Canis) for the serological diagnosis of canine visceral leishmaniasis. Vet Parasitol.

[26] Viviano KR, VanderWielen B (2013). Effect of N-acetylcysteine supplementation on intracellular glutathione, urine isoprostanes, clinical score, and survival in hospitalized ill dogs. J Vet Intern Med.

[27] Issels RD, Nagele A, Eckert KG, Wilmanns W (1988). Promotion of cystine uptake and its utilization for glutathione biosynthesis induced by cysteamine and N-acetylcysteine. Biochem Pharmacol.

[28] Borghi SM, Fattori V, Conchon-Costa I, Pinge-Filho P, Pavanelli WR, Verri WA (2017). Leishmania infection: painful or painless?. Parasitol Res.

[29] Verri WA, Cunha TM, Parada CA, Poole S, Cunha FQ, Ferreira SH (2006). Hypernociceptive role of cytokines and chemokines: targets for analgesic drug development?. Pharmacol Ther.

[30] Woolf CJ, Allchorne A, Safieh-Garabedian B, Poole S (1997). Cytokines, nerve growth factor and inflammatory hyperalgesia: the contribution of tumour necrosis factor alpha. Br J Pharmacol.

[31] Leitl MD, Potter DN, Cheng K, Rice KC, Carlezon WA, Negus SS (2014). Sustained pain-related depression of behavior: effects of intraplantar formalin and complete freund's adjuvant on intracranial self-stimulation (ICSS) and endogenous kappa opioid biomarkers in rats. Mol Pain.

[32] Raghavendra V, Tanga FY, DeLeo JA (2004). Complete Freunds adjuvant-induced peripheral inflammation evokes glial activation and proinflammatory cytokine expression in the CNS. Eur J Neurosci.

[33] Borghi SM, Fattori V, Ruiz-Miyazawa KW, Miranda-Sapla MM, Casagrande R, Pinge-Filho P, Pavanelli WR, Verri WA (2017). Leishmania (L). Amazonensis induces hyperalgesia in balb/c mice: contribution of endogenous spinal cord TNFalpha and NFkappaB activation. Chem Biol Interact.

[34] da Silva SS, Mizokami SS, Fanti JR, Miranda MM, Kawakami NY, Teixeira FH, Araujo EJ, Panis C, Watanabe MA, Sforcin JM (2016). Propolis reduces Leishmania amazonensis-induced inflammation in the liver of BALB/c mice. Parasitol Res.

[35] Cangussu SD, Souza CC, Castro MS, Vieira LQ, Cunha FQ, Afonso LC, Arantes RM (2013). The endogenous cytokine profile and nerve fibre density in mouse ear Leishmania major-induced lesions related to nociceptive thresholds. Exp Parasitol.

[36] Cunha TM, Verri WA, Silva JS, Poole S, Cunha FQ, Ferreira SH (2005). A cascade of cytokines mediates mechanical inflammatory hypernociception in mice. Proc Natl Acad Sci U S A.

[37] Mauel J, Ransijn A (1997). Leishmania spp.: mechanisms of toxicity of nitrogen oxidation products. Exp Parasitol.

[38] Bisti S, Konidou G, Boelaert J, Lebastard M, Soteriadou K (2006). The prevention of the growth of Leishmania major progeny in BALB/c iron-loaded mice: a process coupled to increased oxidative burst, the amplitude and duration of which depend on initial parasite developmental stage and dose. Microbes Infect.

[39] Morabito R, Remigante A, Cavallaro M, Taormina A, La Spada G, Marino A (2017). Anion exchange through band 3 protein in canine leishmaniasis at different stages of disease. Pflugers Arch.

[40] Aruoma OI, Grootveld M, Bahorun T (2006). Free radicals in biology and medicine: from inflammation to biotechnology. Biofactors.

[41] de Menezes JP, Guedes CE, Petersen AL, Fraga DB, Veras PS (2015). Advances in development of new treatment for Leishmaniasis. Biomed Res Int.

[42] Kaplum V, Cogo J, Sangi DP, Ueda-Nakamura T, Correa AG, Nakamura CV (2016). In vitro and in vivo activities of 2,3-Diarylsubstituted Quinoxaline derivatives against Leishmania amazonensis. Antimicrob Agents Chemother.

[43] Rocha-Vieira E, Ferreira E, Vianna P, De Faria DR, Gaze ST, Dutra WO, Gollob KJ (2003). Histopathological outcome of Leishmania major-infected BALB/c mice is improved by oral treatment with N-acetyl-l-cysteine. Immunol.

[44] Keithley EM, Canto C, Zheng QY, Wang X, Fischel-Ghodsian N, Johnson KR (2005). Cu/Zn superoxide dismutase and age-related hearing loss. Hear Res.

[45] Gasparotto J, Kunzler A, Senger MR, Souza CD, Simone SG, Bortolin RC, Somensi N, Dal-Pizzol F, Moreira JC, Abreu-Silva AL (2017). N-acetyl-cysteine inhibits liver oxidative stress markers in BALB/c mice infected with Leishmania amazonensis. Mem Inst Oswaldo Cruz.

[46] Aruoma OI, Halliwell B, Hoey BM, Butler J (1989). The antioxidant action of N-acetylcysteine: its reaction with hydrogen peroxide, hydroxyl radical, superoxide, and hypochlorous acid. Free Radic Biol Med.

[47] Peterson JD, Herzenberg LA, Vasquez K, Waltenbaugh C (1998). Glutathione levels in antigen-presenting cells modulate Th1 versus Th2 response patterns. Proc Natl Acad Sci U S A.

[48] Monteiro MC, Marques FC, Blazius RD, Santos da Silva O, de Queiroz Cunha F, Bento DB, Torres Romao PR (2008). N-acetyl-L: -cysteine reduces the parasitism of BALB/c mice infected with Leishmania amazonensis. Parasitol Res.

[49] Cuzzocrea S, Mazzon E, Esposito E, Muia C, Abdelrahman M, Di Paola R, Crisafulli C, Bramanti P, Thiemermann C (2007). Glycogen synthase kinase-3beta inhibition attenuates the development of ischaemia/reperfusion injury of the gut. Intensive Care Med.

[50] Hargreaves K, Dubner R, Brown F, Flores C, Joris J (1988). A new and sensitive method for measuring thermal nociception in cutaneous hyperalgesia. Pain.

[51] Gugliandolo E, Fusco R, D'Amico R, Militi A, Oteri G, Wallace JL, Di Paola R, Cuzzocrea S (2018). Anti-inflammatory effect of ATB-352, a H2S -releasing ketoprofen derivative, on lipopolysaccharide-induced periodontitis in rats. Pharmacol Res.

[52] Petrosino S, Campolo M, Impellizzeri D, Paterniti I, Allara M, Gugliandolo E, D'Amico R, Siracusa R, Cordaro M, Esposito E (2017). 2-Pentadecyl-2-Oxazoline, the Oxazoline of pea, Modulates Carrageenan-Induced Acute Inflammation. Front Pharmacol.

[53] Salvemini D, Wang ZQ, Wyatt PS, Bourdon DM, Marino MH, Manning PT, Currie MG (1996). Nitric oxide: a key mediator in the early and late phase of carrageenan-induced rat paw inflammation. Br J Pharmacol.

[54] Gu M, A-b Z, Jin J, Cui Y, Zhang N, Z-p C, Wang Y, Zhan J, W-j T. Cardioprotective effects of genistin in rat myocardial ischemia-reperfusion injury studies by regulation of P2X7/NF-κB pathway. Evid Based Complement Alternat Med. 2016;2016. 10.1155/2016/5381290 PMC4818796 27087823

[55] D'amico R, Fusco R, Gugliandolo E, Cordaro M, Siracusa R, Impellizzeri D, Peritore AF, Crupi R, Cuzzocrea S, Di Paola R (2019). Effects of a new compound containing Palmitoylethanolamide and Baicalein in myocardial ischaemia/reperfusion injury in vivo. Phytomedicine.

[56] Ou Z, Zhao J, Zhu L, Huang L, Ma Y, Ma C, Luo C, Zhu Z, Yuan Z, Wu J (2019). Anti-inflammatory effect and potential mechanism of betulinic acid on lambda-carrageenan-induced paw edema in mice. Biomed Pharmacother.

